# Serum uric acid is inversely associated with lung function in US adults

**DOI:** 10.1038/s41598-024-51808-y

**Published:** 2024-01-14

**Authors:** Wen Luo, Chen Wang, Wanyu Wang, Xiangyang Yao, Fang Lu, Dinghui Wu, Yihua Lin

**Affiliations:** 1 Department of Pulmonary and Critical Care Medicine, The First Affiliated Hospital of Xiamen University, School of Medicine, Xiamen University, Xiamen, Fujian People’s Republic of China; 2 Department of Neurology, The First Affiliated Hospital of Xiamen University, School of Medicine, Xiamen University, Xiamen, Fujian People’s Republic of China; 3 Department of Pulmonary, The First Affiliated Hospital of Xiamen University, School of Medicine, Xiamen University, Xiamen, Fujian People’s Republic of China

**Keywords:** Biomarkers, Health care, Medical research

## Abstract

The relationship between serum uric acid and lung function has been controversial. This study aims to determine whether there is an independent relationship between serum uric acid and lung function in the National Health and Nutrition Examination Survey (NHANES) from 2007 to 2012. Serum uric acid was considered the exposure variable, and lung function (FEV1 and FVC) was the outcome variable. Multivariable linear regression was conducted with adjustments for potential confounders. The total number of participants from NHANES (2007–2012) was 30,442, of which 7514 were included in our analysis after applying exclusion criteria. We observed that serum uric acid was negatively associated with FEV1 and FVC after adjusting for confounders (β for FEV1 [− 24.77 (− 36.11, − 13.43)] and FVC [− 32.93 (− 47.42, − 18.45)]). Similarly, serum uric acid showed a negative correlation with FEV1 and FVC after adjusting for confounding variables both in male and female populations. The relationship between serum uric acid and FEV1 and FVC remained consistent and robust in various subgroups within both male and female populations, including age, race, BMI, alcohol consumption, smoking status, and income-poverty ratio. Serum uric acid is negatively associated with FEV1 and FVC in the US general healthy population. This negative relationship is significant in both the male and female populations.

## Introduction

Lung function gradually decreases over time and varies greatly among individuals^1^. Individuals with impairment of lung function are more likely to suffer from chronic respiratory diseases and have an increased risk of all-cause death^2^. Therefore, it is of particular interest to identify biomarkers associated with this impairment^3^.

Uric acid (UA) is the final decomposition product of purine degradation and is present in the plasma and epithelial lining fluid of the respiratory tract^4,5^. Previous studies have indicated that serum UA is associated with cardiovascular diseases, including hypertension, stroke, coronary heart disease, and congestive heart failure^6–9^. Similarly, there is also a variety of research investigating the relationship between serum UA and respiratory disease, including chronic obstructive pulmonary disease (COPD), pulmonary hypertension and obstructive sleep apnea. Significant correlation has been found^10–12^.

Serum UA has been reported to be linked to lung function, but controversial results have been observed in an epidemiological setting: a few studies have shown a negative association between serum UA and lung function in the general healthy population^13–15^. Meanwhile, only in the female population, the negative association between serum UA and lung function was found in the study by Jeong et al.^16^. Nevertheless, a positive relationship between serum UA and lung function was observed in a healthy Korean population^17^. Consequently, this study aimed to investigate the association between serum UA and lung function in the US population using data from the National Health and Nutrition Examination Survey (NHANES 2007-2012).

## Methods

### Population research

Data was obtained from the National Health and Nutrition Examination Survey (NHANES) (2007–2012). NHANES is a complex, stratified, multistage probability sample survey of the non-institutionalized US population. These cross-sectional surveys are used to assess the severity and prevalence of various diseases, as well as to explore potential new directions for medical research and public health policies.

### Inclusion and exclusion criteria

**Figure 1:**
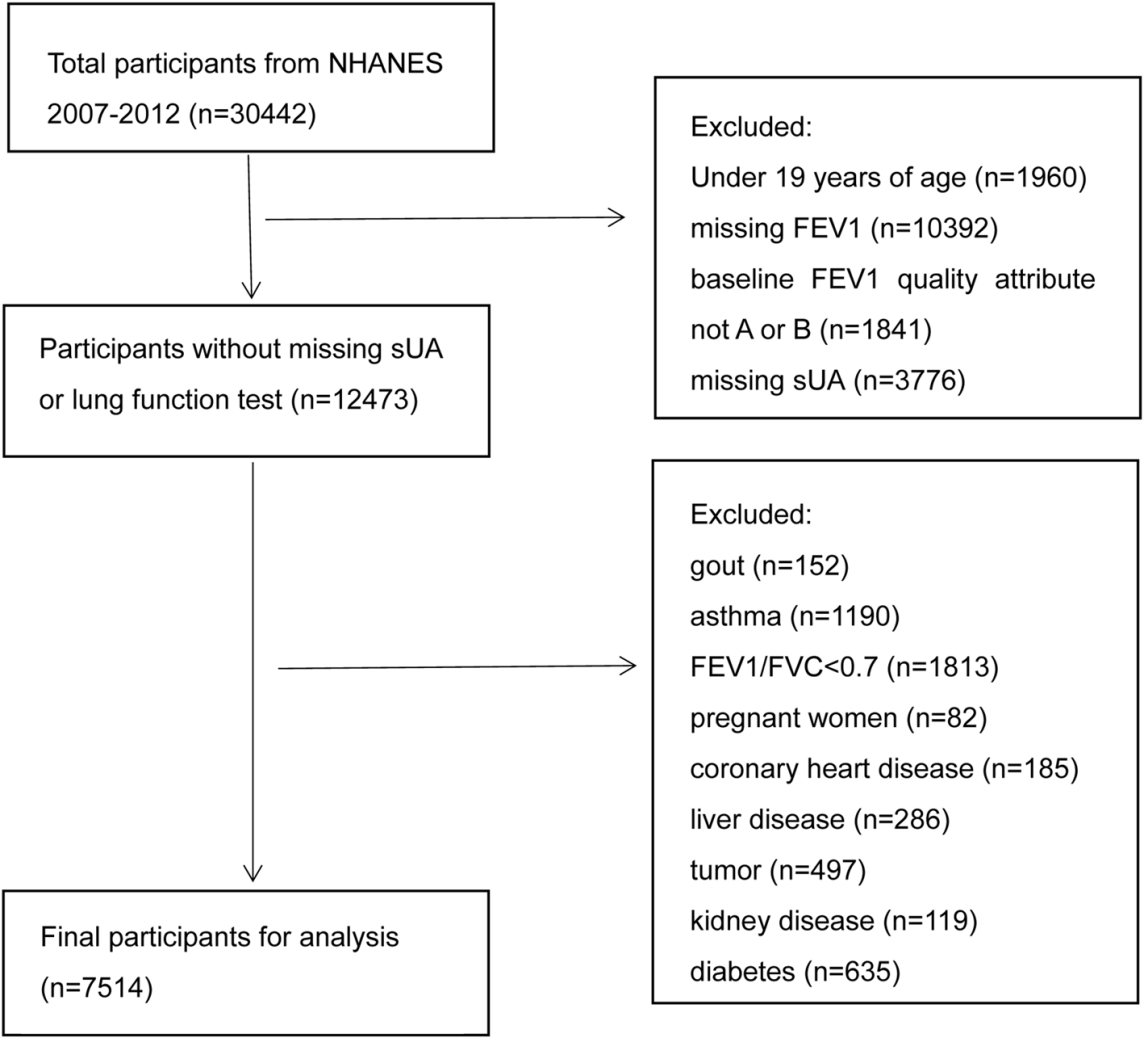
Flowchart of the inclusion of participants.

The population included participants with complete data on serum UA and lung function (specifically forced expiratory volume in one second (FEV1) and forced vital capacity (FVC)). The total number of participants from NHANES (2007–2012) was 30,442. After excluding subjects under 19 years old (n = 1960), those with missing FEV1 (n = 10,392), baseline FEV1 Quality Attribute not A or B (n = 1841), missing serum UA (n = 3776), gout (n = 152), asthma (n = 1190), FEV1/FVC < 0.7 (n = 1813), pregnant women (n = 82), coronary heart disease (n = 185), liver disease (n = 286), tumor (n = 497), kidney disease (n = 119), and diabetes (n = 635), a total of 7514 subjects remained for the final analysis (Fig. 1).

### Ethical approval

Participants aged ≥ 18 years furnished informed consent on their own. The NCHS Ethics Review Board approved the conduct of NHANES, and written informed consent was obtained from all participants.

### Study variables

The principal variables of this study were lung function (dependent variable) and serum UA (independent variable). Serum UA was measured using a Beckman Synchron LX20 (Beckman Coulter, Inc., Brea, CA). Lung function was measured using Ohio 822/827 dry-rolling seal volume spirometers.

The following covariates were included: age, gender, race, income-poverty ratio, body mass index, alcohol drinking, smoke, systolic blood pressure, diastolic blood pressure, blood urea nitrogen, creatinine, calcium, total cholesterol, total protein, fractional exhaled nitric oxide (FeNO) and total bilirubin. Details regarding the measurement process of serum UA and lung function, as well as the acquisition process for other covariates, are available at www.cdc.gov/nchs/nhanes.

### Statistical analyses

NHANES sample weights were taken into account when calculating all estimates. Continuous variables are presented as mean ± standard deviation (SD) or medians (25th percentile–75th percentile), while categorical variables are presented as frequency (%). This research utilized chi-square tests for categorical variables and linear regression models for continuous variables to assess significant differences in these variables. Dummy variables were used to indicate missing covariate values. After adjusting for confounders, multivariable linear regression models were constructed to determine the independent relationship between serum UA and lung function. Generalized additive models and smooth curve fittings were employed to evaluate any non-linear relationship between serum UA and lung function. Stratified and interaction analyses were performed to assess whether covariates influenced the association between serum UA and lung function, ensuring the robustness of data analysis. A 2-tailed *P* < 0.05 was considered statistically significant for all analyses. Data analysis was conducted using the statistical software packages R (http://www.R-project.org) and EmpowerStats (http://www.empowerstats.com).

## Results

### Baseline characteristics of selected participants

A total of 7514 participants were included in the final analysis. The mean age was 40.85 ± 14.28 years old. The mean serum UA levels were 5.34 ± 1.34 mg/dl. Table 1 presents the weighted baseline characteristics according to the serum UA tertiles. All variables showed statistically significant differences among the different serum uric acid groups. Except for the income-poverty ratio, race, and female gender, all other variables exhibited an increasing trend according to the tertiles of serum uric acid (Table 1).

**Table 1:** Description of 7514 participants included in the present study.

Characteristic	All(n = 7514)	Tertile of serum uric acid (mg/dl)			*P* value
		Tertile 1 (< 4.70) (n = 2498)	Tertile 2 (4.70–5.70) (n = 2322)	Tertile 3 (> 5.70) (n = 2694)	
Age (years)	40.85 ± 14.28	40.26 ± 13.76	41.12 ± 14.77	41.15 ± 14.32	0.0418
Gender, n (%)					< 0.0001
Male	3690 (49.11)	366 (14.66)	1132 (48.77)	2152 (79.88)	
Female	3824 (50.89)	2132 (85.34)	1190 (51.23)	542 (20.12)	
Race, n (%)					0.0098
Mexican American	732 (9.74)	243 (9.73)	242 (10.43)	248 (9.21)	
Other Hispanic	458 (6.09)	177 (7.07)	152 (6.54)	131 (4.85)	
Non-Hispanic White	5009 (66.66)	1650 (66.05)	1496 (64.42)	1858 (68.97)	
Non-Hispanic Black	793 (10.55)	261 (10.46)	261 (11.25)	271 (10.07)	
Other Race	524 (6.97)	167 (6.69)	171 (7.36)	186 (6.90)	
Income-poverty ratio, n (%)					0.0081
Poor	1530 (20.36)	522 (20.89)	523 (22.54)	489 (18.17)	
Nearly poor	606 (8.06)	189 (7.56)	191 (8.22)	226 (8.38)	
Not poor	4929 (65.60)	1643 (65.76)	1464 (63.04)	1818 (67.47)	
Missing	449 (5.98)	145 (5.79)	144 (6.19)	161 (5.98)	
Smoke, n (%)					< 0.0001
Yes	2888 (38.44)	879 (35.19)	902 (38.83)	1105 (41.01)	
No	4449 (59.21)	1566 (62.71)	1352 (58.23)	1533 (56.89)	
Missing	177 (2.35)	52 (2.10)	68 (2.94)	57 (2.10)	
Alcohol drinking, n (%)					< 0.0001
Yes	5620 (74.80)	1751 (70.10)	1708 (73.57)	2153 (79.93)	
No	1319 (17.56)	521 (20.86)	444 (19.11)	361 (13.40)	
Missing	574 (7.64)	226 (9.04)	170 (7.31)	180 (6.67)	
Body Mass Index (kg/m^2^)	28.18 ± 6.30	25.94 ± 5.44	28.31 ± 6.13	30.08 ± 6.49	< 0.0001
SBP (mmHg)	119.34 ± 14.65	116.06 ± 14.79	119.54 ± 14.41	122.09 ± 14.12	< 0.0001
DBP (mmHg)	71.26 ± 11.26	69.76 ± 10.67	70.75 ± 10.88	73.00 ± 11.82	< 0.0001
Calcium (mg/dL)	9.42 ± 0.34	9.36 ± 0.34	9.43 ± 0.34	9.47 ± 0.34	< 0.0001
Total bilirubin (mg/dL)	0.77 ± 0.30	0.69 ± 0.26	0.75 ± 0.29	0.85 ± 0.33	< 0.0001
Total Protein (g/dL)	7.15 ± 0.44	7.08 ± 0.45	7.15 ± 0.43	7.21 ± 0.43	< 0.0001
Total cholesterol (mg/dL)	196.51 ± 39.49	194.20 ± 39.26	194.05 ± 39.30	200.50 ± 39.50	< 0.0001
Creatinine (mg/dL)	0.85 ± 0.18	0.74 ± 0.15	0.85 ± 0.16	0.95 ± 0.17	< 0.0001
Blood urea nitrogen (mg/dL)	12.30 ± 4.01	11.24 ± 3.62	12.34 ± 4.07	13.20 ± 4.06	< 0.0001
Serum UA (mg/dl)	5.34 ± 1.34	3.91 ± 0.54	5.19 ± 0.32	6.73 ± 0.82	< 0.0001
FeNO (ppb)	13.50 (9.00–19.50)	12.50 (8.50–17.50)	13.50 (9.00–19.50)	14.00 (9.50–20.50)	< 0.001
FVC (ml)	4248.72 ± 1054.30	3816.56 ± 818.60	4206.57 ± 1090.78	4664.66 ± 1049.27	< 0.0001
FEV1 (ml)	3436.98 ± 863.91	3113.14 ± 679.10	3399.32 ± 898.03	3753.45 ± 870.25	< 0.0001

Data are presented as n (%), means ± SD or medians (25th percentile-75th percentile). BMI: body mass index; SBP: Systolic blood pressure; DBP: Diastolic blood pressure; FEV1: forced expiratory volume in 1 s; FVC: forced vital capacity; UA: uric acid; FeNO: fractional exhaled nitric oxide.

### Association between serum UA and lung function (FEV1 and FVC) in the total population

**Figure 2:**
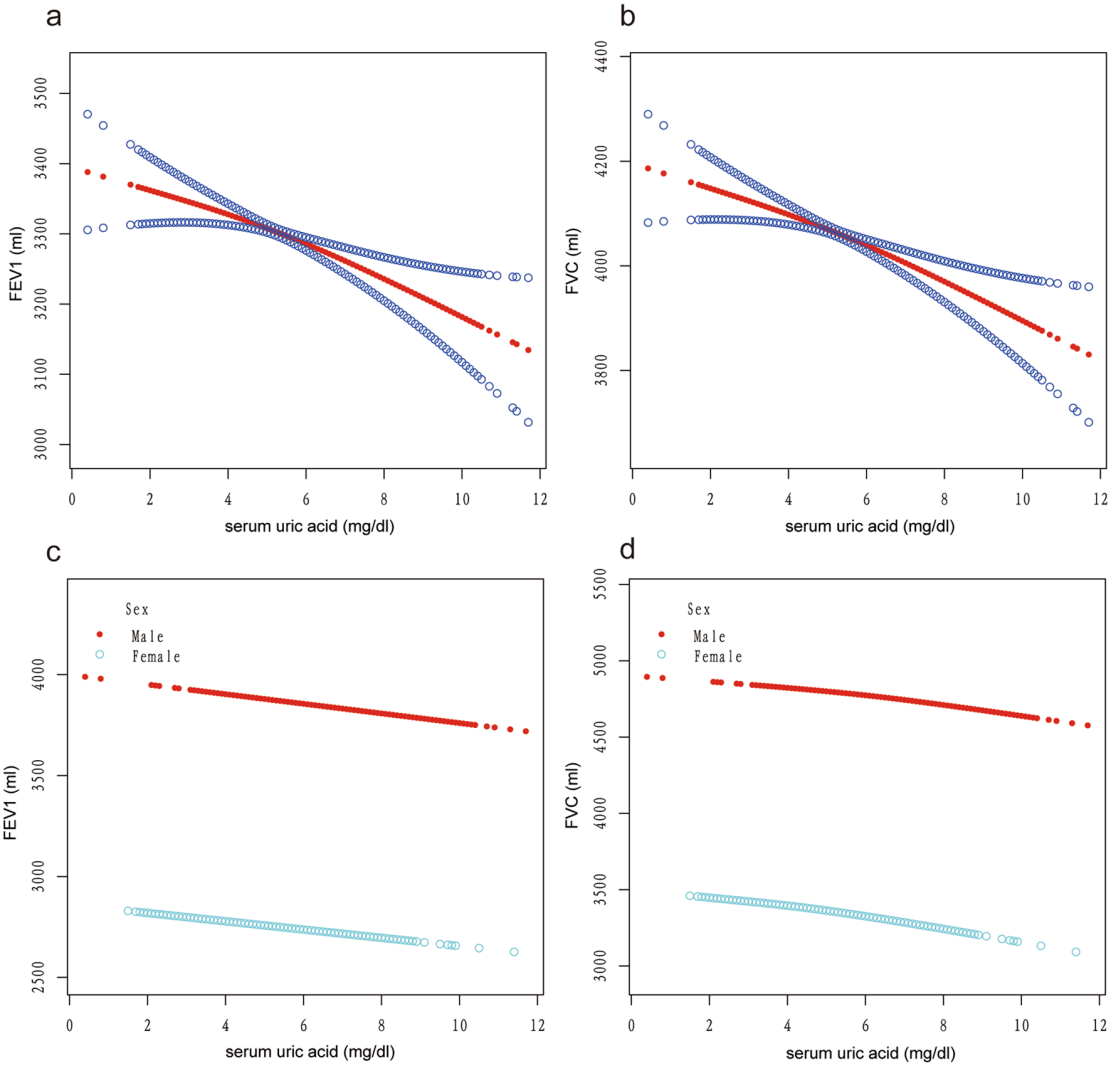
**Correlation between serum uric acid and FEV1 (a and b) and FVC (c and d) in the male and female population.** Correlation between serum uric acid and lung function test (FEV1 and FVC) in the total population (a and b) and stratified by gender (c and d). Each black point represents a sample. The area between two blue dotted lined is expressed as a 95% CI. Each point shows the magnitude of the serum uric acid and is connected to form a continuous line. Age, gender, race, income-poverty ratio, BMI, alcohol drinking, smoke, systolic blood pressure, diastolic blood pressure, blood urea nitrogen, total cholesterol, creatinine, calcium, total protein, FeNO and total bilirubin were adjusted.

The relationship between serum UA and lung function was explored using a generalized additive model. The adjusted smoothed plots indicated a linear relationship between serum UA and lung function both in the total population and male or female populations (Fig. 2). Three models were constructed to analyze the independent role of serum UA in FEV1 and FVC in different populations: the general population, the male population, and the female population. Model 1 involved no modification variables, Model 2 included adjustments for gender or age and race, and Model 3 incorporated adjustments for the covariates presented in Table 1 (Tables 2 and 3). In the general population, Model 1 revealed a positive association between serum UA and FEV1 and FVC, whereas Models 2 and 3 identified a negative association between serum UA and FEV1 and FVC. Model 3 showed a significant negative association between serum UA and FEV1 (β = − 24.77; 95% CI − 36.11 to − 13.43) and FVC (β = − 32.93; 95% CI − 47.42 to − 18.45) (Table 2). Each 1 mg/dl increase in serum UA was associated with a 24.77 ml decline in FEV1 and a 32.93 ml decline in FVC. Sensitivity analysis using tertiles of serum UA as a categorical variable yielded similar results, with negative effect sizes for tertiles 2 and 3 in Models 2 and 3. The *p*-values for trend in all models were significant (all *p* < 0.05) (Table 2). In both the male and female populations, except for a small magnitude positive β value in Model 1 for FVC in males (with a *p*-value > 0.05), all other models showed negative effect sizes. Furthermore, all p-values in Model 3 were significant, indicating a negative relationship between serum UA and lung function in both male and female populations. When serum UA was categorized into tertiles as a categorical variable, the results remained consistent with the findings obtained when serum UA was treated as a continuous variable (Table 3).

**Table 2:** Association of serum uric acid with lung function in different models in general population.

Variable	Model 1 (n = 7514)		Model 2 (n = 7514)		Model 3 (n = 7514)	
	β (95% CI)	*P*-value	β (95% CI)	*P*-value	β (95% CI)	*P*-value
For FEV1 (ml)						
UA (mg/dl)	201.50 (187.60, 215.41)	< 0.0001	− 45.71 (− 58.75, − 32.68)	< 0.0001	− 24.77 (− 36.11, − 13.43)	< 0.0001
UA (mg/dl) in tertile						
Tertile 1	Reference		Reference		Reference	
Tertile 2	286.18 (239.18, 333.18)	< 0.0001	− 127.94 (− 166.72, − 89.16)	0.0001	− 47.08 (− 77.48, − 16.67)	0.0024
Tertile 3	640.31 (596.01, 684.60)	< 0.0001	− 151.41 (− 192.96, − 109.86)	0.0001	− 59.93 (− 94.77, − 25.09)	0.0008
*P* for trend		< 0.001		< 0.001		< 0.001
For FVC (ml)						
UA (mg/dl)	263.97 (247.14, 280.80)	< 0.0001	− 49.11 (− 64.42, − 33.81)	< 0.0001	− 32.93 (− 47.42, − 18.45)	< 0.0001
UA (mg/dl) in tertile						
Tertile 1	Reference		Reference		Reference	
Tertile 2	390.01 (333.20, 446.83)	< 0.0001	− 132.38 (− 177.95, − 86.82)	0.0001	− 49.04 (− 87.87, − 10.20)	0.0134
Tertile 3	848.11 (794.56, 901.65)	< 0.0001	− 150.61 (− 199.43, − 101.79)	< 0.0001	− 73.29 (− 117.79, − 28.78)	0.0013
*P* for trend		< 0.001		< 0.001		0.001

Model 1, no covariates were adjusted.

Model 2, gender was adjusted.

Model 3, Age, gender, race, income-poverty ratio, BMI, alcohol drinking, smoke, systolic blood pressure, diastolic blood pressure, blood urea nitrogen, total cholesterol, creatinine, calcium, total protein, FeNO and total bilirubin were adjusted.

FEV1: forced expiratory volume in 1 s; FVC: forced vital capacity; UA: uric acid.

**Table 3:** Association of serum uric acid with lung function in different models in male and female population.

Variable	Model 1		Model 2		Model 3	
	β (95% CI)	*P*-value	β (95% CI)	*P*-value	β (95% CI)	*P*-value
For FEV1 (ml) in male (n = 3684 in each model)						
UA (mg/dl)	− 0.91 (− 20.86, 19.04)	0.9285	− 16.63 (− 32.43, − 0.83)	0.0392	− 24.07 (− 41.37, − 6.77)	0.0064
UA (mg/dl) in tertile						
Tertile 1	Reference		Reference		Reference	
Tertile 2	10.11 (− 74.44, 94.67)	0.8147	− 53.68 (− 120.54, 13.17)	0.1156	− 66.51 (− 132.55, − 0.46)	0.0485
Tertile 3	19.77 (− 59.06, 98.60)	0.6231	− 51.83 (− 114.34, 10.67)	0.1042	− 74.38 (− 138.38, − 10.39)	0.0228
For FVC (ml) in male (n = 3684 in each model)						
UA (mg/dl)	1.36 (− 22.42, 25.14)	0.9107	− 19.31 (− 39.58, 0.95)	0.0618	− 30.06 (− 52.26, − 7.85)	0.0080
UA (mg/dl) in tertile						
Tertile 1	Reference		Reference		Reference	
Tertile 2	14.07 (− 86.72, 114.85)	0.7844	− 58.74 (− 144.50, 27.01)	0.1795	− 68.86 (− 153.66, 15.93)	0.1115
Tertile 3	41.38 (− 52.58, 135.34)	0.3881	− 54.72 (− 134.89, 25.46)	0.1811	− 82.29 (− 164.45, − 0.12)	0.0497
For FEV1 (ml) in female (n = 3830 in each model)						
UA (mg/dl)	− 94.31 (− 110.91, − 77.72)	< 0.0001	− 32.93 (− 45.31, − 20.55)	< 0.0001	− 32.57 (− 46.96, − 18.17)	< 0.0001
UA (mg/dl) in tertile						
Tertile 1	Reference		Reference		Reference	
Tertile 2	− 146.57 (− 187.55, − 105.59)	< 0.0001	− 61.78 (− 91.95, − 31.61)	< 0.0001	− 53.83 (− 85.05, − 22.62)	0.0007
Tertile 3	− 281.94 (− 334.68, − 229.19)	< 0.0001	− 95.76 (− 135.06, − 56.45)	< 0.0001	− 83.52 (− 126.59, − 40.45)	0.0001
For FVC (ml) in female (n = 3830 in each model)						
UA (mg/dl)	− 103.87 (− 122.91, − 84.82)	< 0.0001	− 48.71 (− 64.51, − 32.91)	< 0.0001	− 44.44 (− 62.65, − 26.23)	< 0.0001
UA (mg/dl) in tertile						
Tertile 1	Reference		Reference		Reference	
Tertile 2	− 147.21 (− 194.29, − 100.13)	< 0.0001	− 68.43 (− 106.97, − 29.89)	0.0005	− 51.26 (− 90.76, − 11.75)	0.0110
Tertile 3	− 306.34 (− 366.94, − 245.74)	< 0.0001	− 141.44 (− 191.65, − 91.24)	< 0.0001	− 113.05 (− 167.55, − 58.55)	< 0.0001

Model 1, no covariates were adjusted.

Model 2, Age and race was adjusted.

Model 3, Age, race, income-poverty ratio, BMI, alcohol drinking, smoke, systolic blood pressure, diastolic blood pressure, blood urea nitrogen, total cholesterol, creatinine, calcium, total protein, FeNO and total bilirubin were adjusted.

FEV1: forced expiratory volume in 1 s; FVC: forced vital capacity; UA: uric acid.

### Subgroup analyses

The role of other covariates on the association between serum UA and FEV1 and FVC was further examined in the male and female populations. The association between serum UA and FEV1 and FVC remained consistent and robust in both male and female populations across various subgroups, including age, race, BMI, alcohol drinking, smoke, and income-poverty ratio. Furthermore, all effect sizes of these subgroups were negative, and there was no significant p-interaction (Figs. S1, S2 in the Supplementary Appendix).

## Discussion

The primary objective of this study was to investigate the independent association between serum UA and lung function in healthy US adults. Our findings revealed a negative association between serum UA and lung function (FEV1 and FVC) after adjusting for potential confounding factors. This relationship was consistent both in males and females. Further research is required to explore whether there exists a causal, pathophysiological mechanism linking serum UA and lung function, as well as to determine the potential utility of serum UA as a biomarker for identifying impaired lung function.

The positive relationship between serum UA and lung function was observed when no covariates were adjusted. However, after only adjusting for gender, the association changed to a negative one. Moreover, even after adjusting for other covariates, the negative association between serum UA and lung function remained. The observational analysis found a correlation between high plasma urate levels and low lung function. Furthermore, our study found a robust negative relationship between serum UA and lung function across various subgroups, including age, race, BMI, smoke, and alcohol consumption, in the overall study population. This negative relationship was also observed within the male and female subgroups mentioned above.

Possible explanations for the negative association between serum UA and lung function can be identified as follows. Firstly, the relationship might be attributed to reverse causation. Previous research has indicated that serum UA levels increase in hypoxic conditions, such as chronic heart failure and COPD^18^. It has also been suggested that pulmonary hypoxia triggers the breakdown of purines, resulting in the elevated production of serum UA ^19^. However, it remains uncertain whether the mild hypoxia observed in the general healthy population could impact serum UA levels, as our study excluded individuals with evident clinical conditions such as asthma, airflow limitation, and coronary heart disease. Secondly, the formation of uric acid necessitates the catalytic activity of xanthine oxidase, a process that occurs in the epithelial lining fluid of the respiratory tract and is accompanied by the generation of superoxide^20^. As superoxide is a free radical, it has the potential to induce oxidative damage to biological molecules. Therefore, it can be speculated that lung tissue damage may occur in the presence of high xanthine oxidase activity due to oxidative stress.

Our study makes a valuable contribution to the existing literature by demonstrating a consistent negative association between serum uric acid (UA) and lung function in both male and female individuals in the US general healthy population. Previous studies have attempted to assess the link between serum UA and lung function in the general population, but the results have been inconsistent. For instance, Hong et al. found a negative relationship between hyperuricemia and FEV1% or FVC% in a sample of 2901 participants from the Korean general population^15^. Similar findings were reported by Aida et al. and Kobylecki et al.^13,14^, which align with our own findings. However, Song et al. reported a potential positive effect of serum UA on lung function in middle-aged, healthy populations^17^. We speculate that the discrepancy in results could be attributed to several factors: First, participant exclusion criteria differed between the studies. Song et al. excluded individuals with chronic lung disease or abnormal chest radiograph findings, but the definition of these diseases was vague, and details were not provided. As such, it is challenging to determine the influence of these excluded participants on the overall conclusion. In contrast, our study excluded participants with asthma and FEV1/FVC < 0.7, and in these individuals, a negative relationship between serum UA and lung function was observed^21,22^. Thus, our conclusion is more robust, given the exclusion of these participants. Second, Song et al. did not assess the adjusted non-linear relationship between serum UA and lung function, nor did they conduct subgroup analyses as a sensitivity analysis for their conclusion. In our study, all effect sizes of subgroups were found to be negative, indicating the robustness of our findings. Third, the study populations differed, with our study focusing on US individuals while Song et al. targeted individuals from South Korea. Fourth, compared to our research, Song et al. did not consider the effects of FeNO, total bilirubin, and income-poverty ratio when adjusting for covariates in the relationship between serum UA and lung function. However, previous studies have confirmed that these variables are associated with lung function^23–25^.

Kobylecki et al.^14^ conducted a Mendelian randomisation study to investigate the causal relationship between serum uric acid and lung function. Their observational analysis found a correlation between high plasma urate levels and worse lung function. However, genetically high plasma urate levels did not show a direct causal association with the outcomes. Likely potential explanations include reverse causation and unmeasured confounding factors. It is important to note that the study population mainly consisted of individuals of Danish descent and had specific inclusion and exclusion criteria that significantly differed from our study, as the United States is a multicultural country with diverse ethnicities. Further research is still required to assess the causal relationship between serum UA and lung function.

The study possesses several strengths. Firstly, a nonlinearity assessment was incorporated, providing a more comprehensive understanding of the relationship between serum uric acid (UA) and lung function. Secondly, additional confounding variables such as Fractional exhaled nitric oxide (FeNO), total bilirubin, and income-poverty ratio were adjusted for. Thirdly, various sensitivity and subgroup analyses were performed, and the findings consistently upheld their robustness. Lastly, the study had a relatively large sample size in comparison to previous studies with similar objectives.

The study has several limitations. Firstly, due to its cross-sectional design, a causal relationship between serum uric acid (UA) and lung function cannot be established. Secondly, the use of uric acid-lowering medications was not accounted for, although individuals with gout were excluded. Lastly, the study population was derived from NHANES (2007-2012), and certain exclusion criteria were applied, potentially limiting the generalizability and extrapolation of the findings.

In conclusion, serum uric acid is negatively associated with FEV1 and FVC in the US general healthy population. This negative relationship is significant in both the male and female populations. These outcomes emphasize the significance of serum uric acid as a potential mechanism underlying FEV1 and FVC decline. Further epidemiologic studies will still be required to confirm this reverse association.

### Supplementary Information

Supplementary Figures.

## Data Availability

Data described in the manuscript will not be made available because the data used in this study were from the NHANES database, which is a free and open database for all researchers around the world. The link to the database is https://wwwn.cdc.gov/nchs/nhanes/Default.aspx.

## References

[1] Ortega VE, Kumar R (2015). The effect of ancestry and genetic variation on lung function predictions: What is "normal" lung function in diverse human populations?. Curr. Allergy Asthma Rep..

[2] Mannino DM, Reichert MM, Davis KJ (2006). Lung function decline and outcomes in an adult population. Am. J. Respir. Crit. Care Med..

[3] Baughman P (2012). Combined effect of lung function level and decline increases morbidity and mortality risks. Eur. J. Epidemiol..

[4] van der Vliet A (1999). Determination of low-molecular-mass antioxidant concentrations in human respiratory tract lining fluids. Am. J. Physiol..

[5] Peden DB (1990). Uric acid is a major antioxidant in human nasal airway secretions. Proc. Natl. Acad. Sci. U. S. A..

[6] Li M, Hou W, Zhang X, Hu L, Tang Z (2014). Hyperuricemia and risk of stroke: A systematic review and meta-analysis of prospective studies. Atherosclerosis.

[7] Zuo T (2016). Hyperuricemia and coronary heart disease mortality: A meta-analysis of prospective cohort studies. BMC Cardiovasc. Disord..

[8] Buzas R, Tautu OF, Dorobantu M, Ivan V, Lighezan D (2018). Serum uric acid and arterial hypertension-Data from Sephar III survey. PLoS One.

[9] Xu H, Liu Y, Meng L, Wang L, Liu D (2021). Effect of uric acid-lowering agents on patients with heart failure: A systematic review and meta-analysis of randomised controlled trials. Front. Cardiovasc. Med..

[10] Dhaun N (2014). Endothelin antagonism and uric acid levels in pulmonary arterial hypertension: Clinical associations. J. Heart Lung Transpl..

[11] Wan YF (2014). Uric acid levels in obstructive sleep apnea patients with atrial fibrillation. Arch. Med. Res..

[12] Rumora L (2020). Uric acid and uric acid to creatinine ratio in the assessment of chronic obstructive pulmonary disease: Potential biomarkers in multicomponent models comprising IL-1beta. PLoS One.

[13] Aida Y (2011). The relationship between serum uric acid and spirometric values in participants in a health check: The Takahata study. Int. J. Med. Sci..

[14] Kobylecki CJ, Vedel-Krogh S, Afzal S, Nielsen SF, Nordestgaard BG (2018). Plasma urate, lung function and chronic obstructive pulmonary disease: A Mendelian randomisation study in 114 979 individuals from the general population. Thorax.

[15] Hong JW, Noh JH, Kim DJ (2020). Association between serum uric acid and spirometric pulmonary function in Korean adults: The 2016 Korea National Health and Nutrition Examination Survey. PLoS One.

[16] Jeong H (2021). Gender-specific association of serum uric acid and pulmonary function: Data from the Korea national health and nutrition examination survey. Medicina (Kaunas).

[17] Song JU, Hwang J, Ahn JK (2017). Serum uric acid is positively associated with pulmonary function in Korean health screening examinees. Mod. Rheumatol..

[18] Holme I, Aastveit AH, Hammar N, Jungner I, Walldius G (2009). Uric acid and risk of myocardial infarction, stroke and congestive heart failure in 417,734 men and women in the Apolipoprotein MOrtality RISk study (AMORIS). J. Intern. Med..

[19] Baker JE (2007). Nitrite confers protection against myocardial infarction: Role of xanthine oxidoreductase, NADPH oxidase and K(ATP) channels. J. Mol. Cell Cardiol..

[20] Pinamonti S (1996). Xanthine oxidase activity in bronchoalveolar lavage fluid from patients with chronic obstructive pulmonary disease. Free Radic. Biol. Med..

[21] Li L, Wan C, Wen F (2014). An unexpected role for serum uric acid as a biomarker for severity of asthma exacerbation. Asian Pac. J. Allergy Immunol..

[22] Kahnert K (2018). Uric acid, lung function, physical capacity and exacerbation frequency in patients with COPD: A multi-dimensional approach. Respir. Res..

[23] Hegewald MJ, Crapo RO (2007). Socioeconomic status and lung function. Chest.

[24] Curjuric I (2014). Serum bilirubin is associated with lung function in a Swiss general population sample. Eur. Respir. J..

[25] Coumou H, Westerhof GA, de Nijs SB, Zwinderman AH, Bel EH (2018). Predictors of accelerated decline in lung function in adult-onset asthma. Eur. Respir. J.

