# Supplementary material for: Serum uric acid is inversely associated with lung function in US adults
Source: Sci Rep. 2024 Jan 14;14:1300. doi: 10.1038/s41598-024-51808-y (PMC10788334; doi:10.1038/s41598-024-51808-y)
Supplement: Supplementary file 1 — Supplementary Figures. [file 41598_2024_51808_MOESM1_ESM.docx]

**Supplementary Appendix**

**Figure S1. The association between serum uric acid and FEV1 (a) and FVC (b) in different subgroups of male population.**


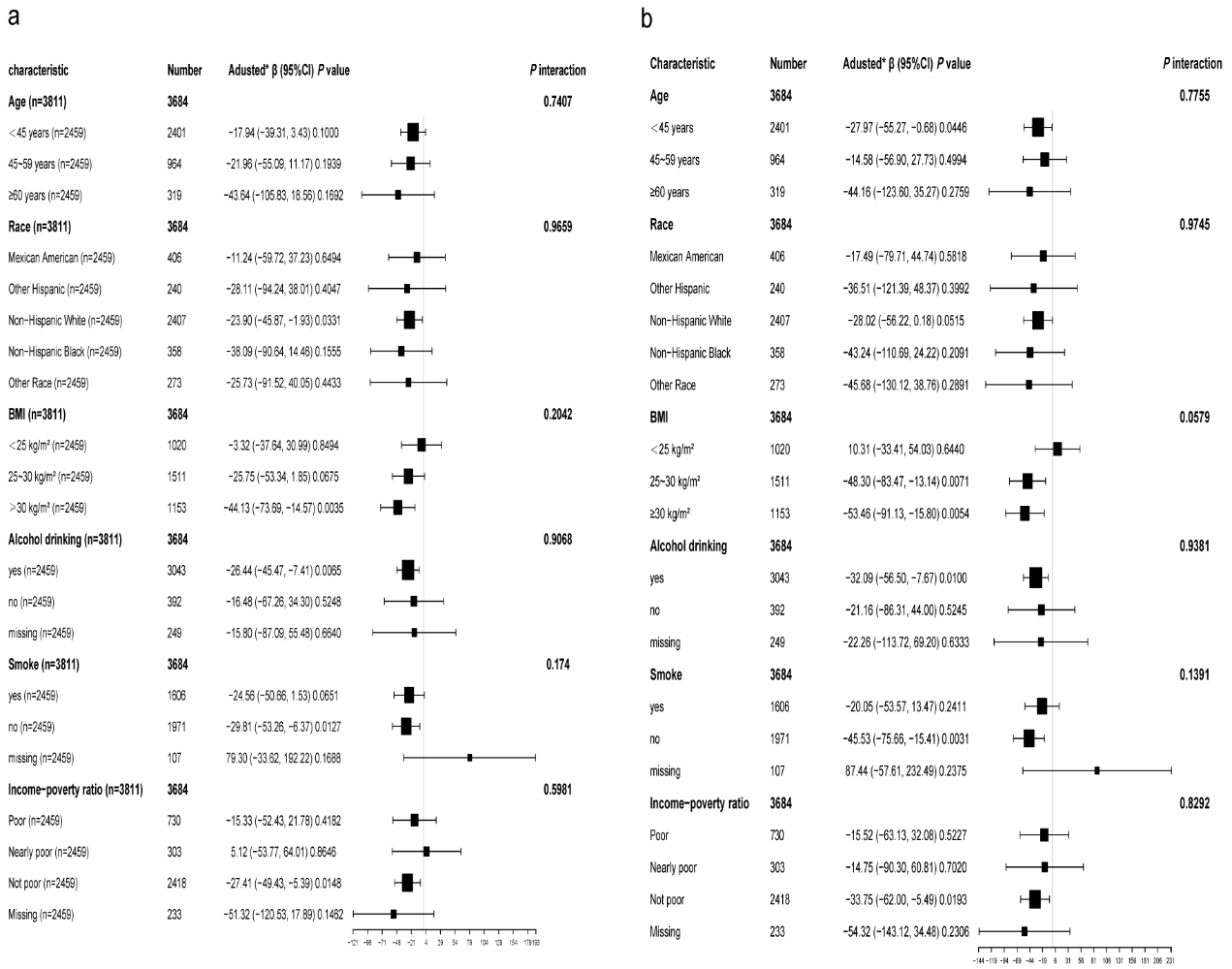


*: Each subgroup analysis adjusted, if not stratified, for age, race, income-poverty ratio, BMI, systolic blood pressure, diastolic blood pressure, blood urea nitrogen, cholesterol, creatinine, total protein, FeNO, calcium, alcohol drinking, smoke and total bilirubin.

**Figure S2. The association between serum uric acid and FEV1 (a) and FVC (b) in different subgroups of female population.**


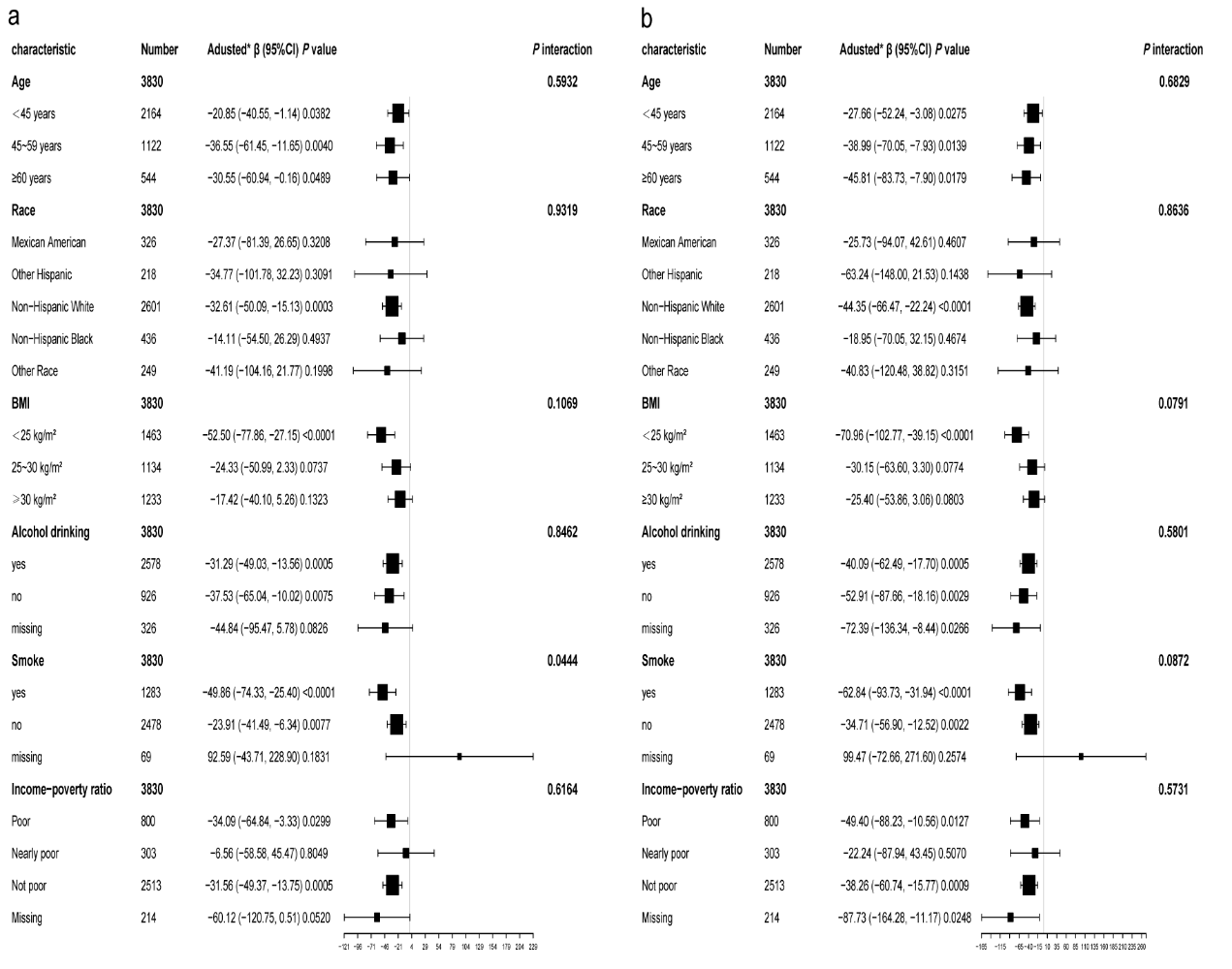


*: Each subgroup analysis adjusted, if not stratified, for age, race, income-poverty ratio, BMI, systolic blood pressure, diastolic blood pressure, blood urea nitrogen, cholesterol, creatinine, total protein, FeNO, calcium, alcohol drinking, smoke and total bilirubin.
